# When Do Ionic Liquids Stop Behaving as Ionic Liquids? Assessing the Environmental Relevance of Ion Pairing

**DOI:** 10.3390/molecules31152619

**Published:** 2026-07-27

**Authors:** Natalia Lisiecka, Marta Woźniak-Karczewska, Anna Parus, Paolo Roccaro, Hermann J. Heipieper, Łukasz Chrzanowski

**Affiliations:** 1 Institute of Chemical Technology and Engineering, Faculty of Chemical Technology, Poznan University of Technology, Berdychowo 7 4, 60-965 Poznan, Poland; marta.wozniak-karczewska@put.poznan.pl (M.W.-K.); anna.parus@put.poznan.pl (A.P.); 2 Department of Molecular Environmental Biotechnology, Helmholtz Centre for Environmental Research—UFZ, Permoserstraße 15, 04318 Leipzig, Germany; hermann.heipieper@ufz.de; 3 Department of Civil Engineering and Architecture, University of Catania, Via Santa Sofia 64, 95123 Catania, Italy; paolo.roccaro@unict.it

**Keywords:** adaptation, adsorption, biodegradation, microorganisms, toxicity, surfactants

## Abstract

Ionic liquids (ILs) have attracted considerable scientific interest over nearly three decades, with thousands of studies highlighting their low volatility, thermal stability, and potential for property modification through cation and anion selection. However, their behavior under environmental conditions remains less clear, particularly when water, soil constituents, and competing ions are present. This article examines whether concepts developed for neat ILs can be directly transferred to aqueous and soil systems. Particular attention is given to ion pairing, dissociation, surfactant-like behavior, sorption, toxicity, and biodegradation. Evidence from studies on herbicidal ILs, conventional precursor salts, and their mixtures indicates that, under environmentally relevant conditions, cations and anions frequently behave as largely independent chemical species. Hydrophobic cations are often responsible for sorption and microbial toxicity, whereas herbicidal anions typically retain mobility and biodegradation patterns comparable to their conventional salt forms. These observations question the environmental significance of ion pairing and suggest that the extent to which IL properties can be controlled through cation and anion selection may be more limited than commonly assumed. Greater emphasis should therefore be placed on precise terminology, appropriate experimental controls, and validation under realistic environmental conditions when interpreting the behavior and environmental relevance of ILs.

## 1. Introduction

**Figure 1:**
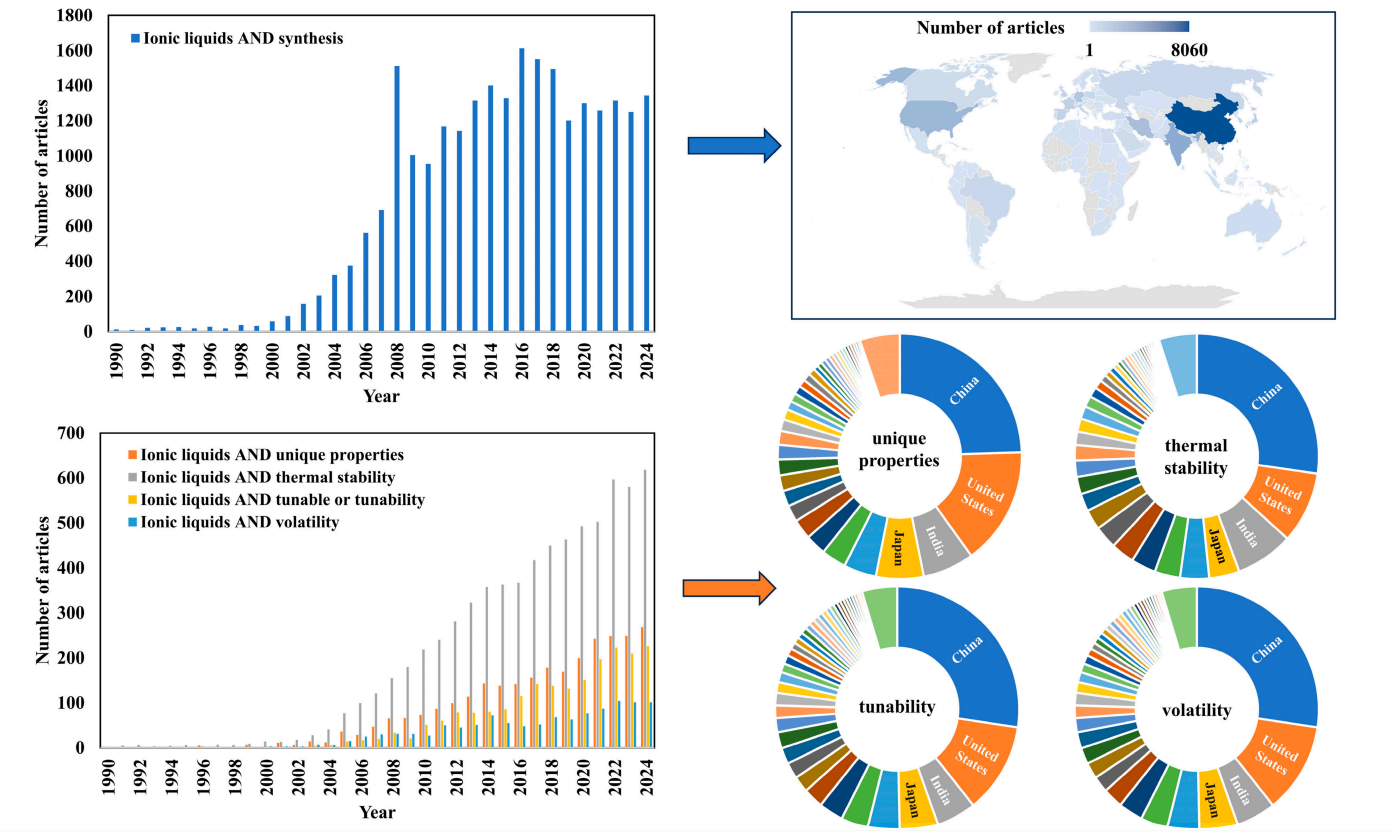
Global trends in ionic liquids research based on a Scopus analysis of publication trends, countries, and frequently associated keywords.

**Figure 2:**
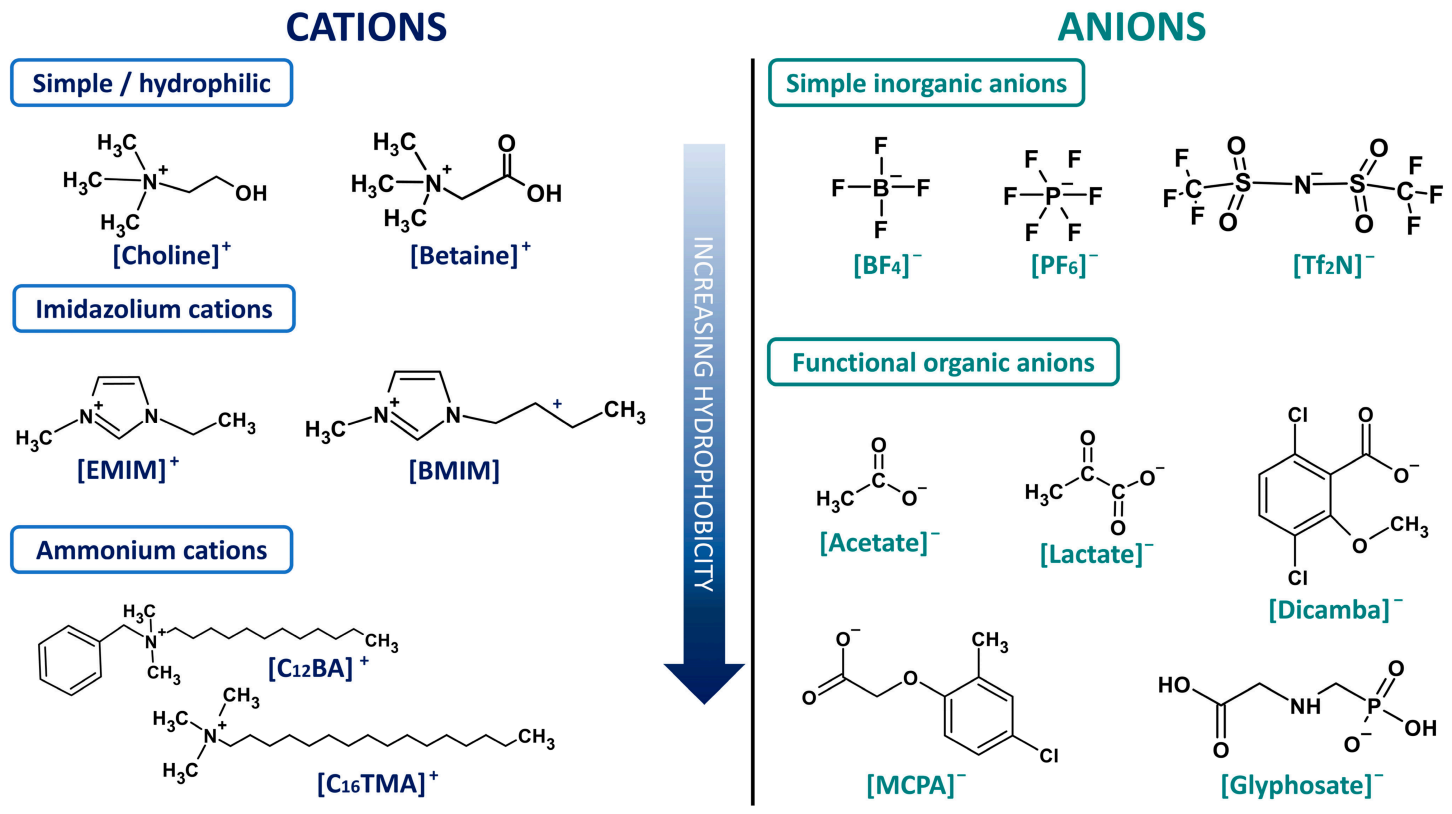
Representative examples of cations and anions commonly investigated in ionic liquid research.

Ionic liquids (ILs) have become an important area of research in modern chemistry [1,2]. Since the first reports describing organic salts that remain liquid at unusually low temperatures, interest in these compounds has grown considerably [3]. Since then, numerous review articles have been published, describing progress in the field [4,5,6,7,8,9]. As with many emerging research areas, initial studies focused on the synthesis of new ILs based on simple organic cations combined with inorganic anions [10,11,12,13], gradually increasing the complexity by introducing organic anions as well [14,15,16,17]. These synthetic efforts were accompanied by analyses of the properties of the resulting ILs [18,19,20,21,22,23,24]. In the next stage, attempts were made to explore their practical applications [25,26]. In parallel, studies examined the effects of ILs on biological systems and later expanded to more complex models, including cell lines [27,28] bacteria [29,30,31,32,33], fungi [34,35,36,37,38], plants [39,40,41,42,43] and other organisms [44,45,46,47,48,49]. Despite substantial progress in environmental and toxicity studies, discussions of ILs continue to focus primarily on their low volatility [50,51,52], high thermal stability [14,53,54], and tunable properties [55,56,57,58] through cation and anion selection (Figure 1). Representative examples of the cations and anions discussed throughout this review are presented in Figure 2.

ILs are widely recognized as green solvents with broad applications in sustainable chemistry and advanced technologies, which has contributed to their growing popularity in scientific research [16,59,60]. Their low or negligible volatility is frequently highlighted as one of their major advantages. However, volatility alone should not be considered a sufficient indicator of environmental safety. Once introduced into environmental systems, ILs may interact with water, soil constituents, microorganisms, plants, and other biological compartments. Consequently, their environmental behavior is influenced by a range of factors beyond volatility, including solubility, hydrophobicity, sorption potential, persistence, and biological activity [16,21,29,30,31,32,33,34,35]. Among these factors, hydrophobicity is particularly important because it influences the interactions of ILs with biological membranes, soil organic matter, and other environmental compartments [21,29,32,33].

One of the most frequently cited advantages of ILs is the possibility of modifying their properties through the selection of specific cations and anions. In many cases, this approach involves combining hydrophobic cations with hydrophilic anions in an attempt to alter properties such as mobility, sorption, toxicity, or environmental persistence [18,19,20,21,22,23,24,55,56,57,58]. Consequently, understanding the role of hydrophobicity becomes essential when evaluating whether such modifications result from interactions between ions or simply reflect the intrinsic properties of the individual cations and anions. This issue is particularly relevant in environmental systems, where water, dissolved ions, and natural sorbents may substantially influence the stability and behavior of ionic species [21,61,62].

**Figure 3:**
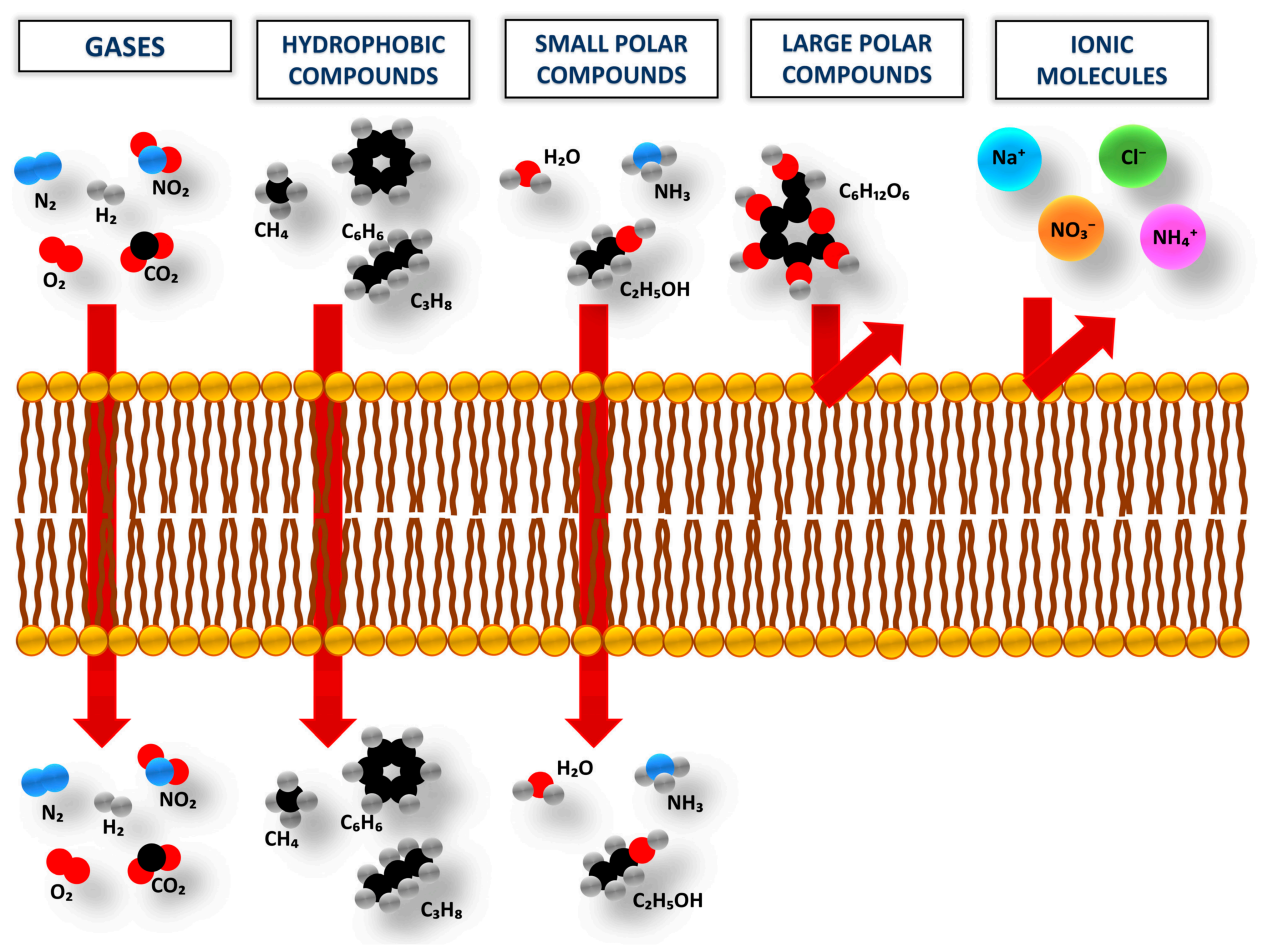
Cell membrane permeability concerning different types of molecules. To observe the interactions of cationic surfactants (such as quaternary ammonium cations) with the membrane, please refer to Figure 4.

In principle, hydrophobic compounds can penetrate biological membranes, provided that they are not electrically charged [63,64,65]. The absence of charge facilitates passive diffusion and accumulation within biological membranes, as illustrated in Figure 3. Through this mechanism, compounds such as hydrocarbons [66] and organometallic substances including dimethylmercury and tetraethyl lead can readily cross biological membranes [67,68]. These compounds do not require auxiliary agents, such as biosurfactants, to facilitate membrane transport [69,70]. In contrast, charged molecules generally require specific transmembrane channels or suitable transport mediators for efficient passage across biological membranes [71,72].

**Figure 4:**
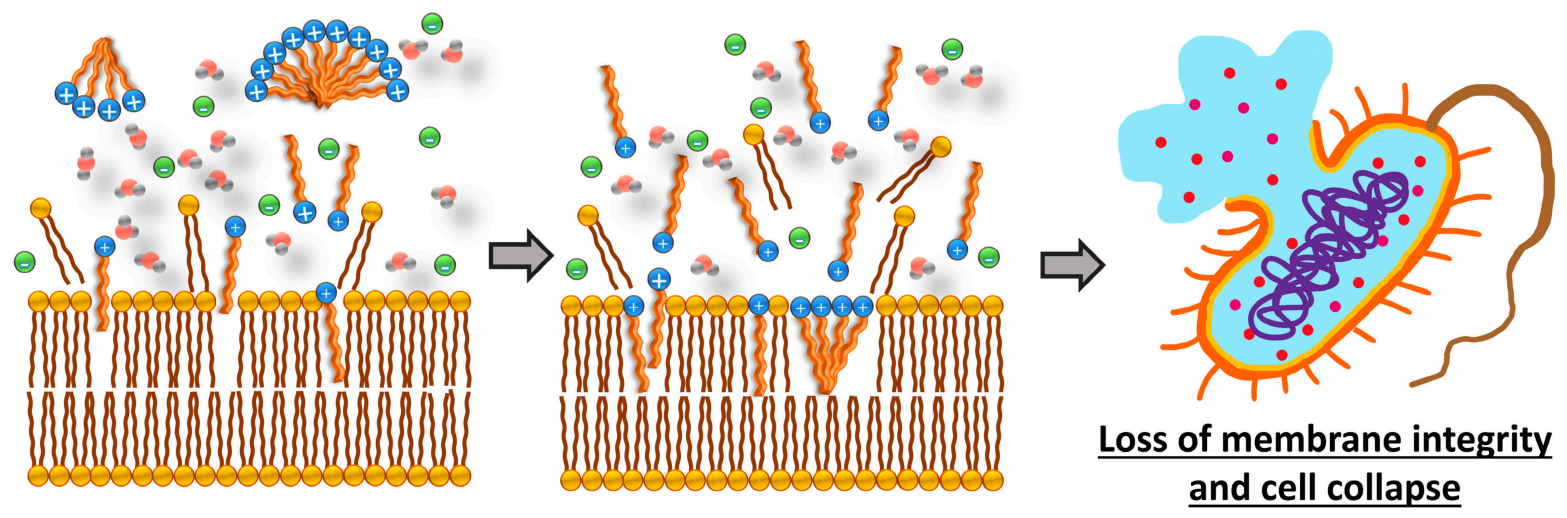
Processes of surfactant-induced disruption of bacterial membrane integrity.

However, if a charged molecule also possesses a hydrophobic tail of appropriate length, it can likewise interact with cell membranes, as shown in Figure 4. Such amphipathic compounds, which combine both hydrophilic and hydrophobic properties within a single molecule, have long been known as surfactants [73]. It is important to note, however, that their activity requires an aqueous environment. Despite the central role of water in environmental systems, the implications of this fact for the behavior of ILs are not always explicitly considered. This raises an important question regarding stability and relevance of ionic interactions once water becomes the dominant component of the system.

What happens to ionic interactions when water is introduced into the system? This is a crucial question, as water can promote ion dissociation, alter solvation processes, and influence the reactivity and mobility of ionic species. To what extent do ILs preserve their characteristic ion-pair interactions under such conditions? Are their properties still governed by interactions between cations and anions, or do the individual properties of the constituent ions become the dominant factor? How stable are ion pairs in aqueous and soil environments, and to what extent do they influence the environmental behavior of ILs?

The questions outlined above remain central to the ongoing discussion regarding the environmental relevance of ILs. Particular attention should be given to the stability of ion pairs, the extent to which IL properties can be controlled through cation and anion selection, and the interpretation of results obtained in aqueous and soil systems. Evidence from studies on sorption, toxicity, biodegradation, and environmental fate allows a critical re-examination of whether concepts developed for neat ILs remain applicable once these compounds are introduced into complex environmental matrices. Addressing these issues is essential for improving the interpretation of experimental observations and for guiding future research toward environmentally relevant systems and rigorous validation of commonly accepted assumptions.

In this review, the question of when ionic liquids stop behaving as ionic liquids is considered from an environmental rather than a formal chemical perspective. The discussion does not concern whether a compound fulfills the physicochemical definition of an ionic liquid, but whether its environmental behavior continues to be governed by interactions between the cation and anion after introduction into environmentally relevant aqueous and soil systems. Throughout this review, the expression “behaving as ionic liquids” therefore refers to situations in which persistent cation–anion interactions remain the dominant factor controlling properties such as sorption, toxicity, biodegradation, mobility, and environmental fate. Conversely, if these properties are determined primarily by the independent behavior of the constituent ions following solvation, the practical environmental relevance of treating the system as an intact ionic liquid becomes questionable. For this reason, the discussion is intentionally restricted to environmentally relevant aqueous and soil systems, where solvation, competing ions, and natural matrices govern ion behavior, rather than to neat ionic liquids or highly concentrated ionic liquid mixtures, in which ion pairing and supramolecular organization may remain dominant.

## 2. Main Body

### 2.1. Ionic Liquids in the Context of Conventional Ionic Compounds

Sodium chloride (NaCl) is one of the most widely recognized examples of a compound stabilized by ionic interactions (Figure 4). Its crystal lattice consists of oppositely charged ions arranged to maximize electrostatic interactions and structural stability [74]. As a result, the crystal structure is highly ordered and maintained by strong attractive forces between neighboring ions. The disruption of this structure may occur through either melting or dissolution, both of which involve weakening the interactions responsible for lattice stability. Understanding these processes provides a useful basis for discussing the behavior of ILs and the nature of ionic interactions in more complex systems.

In the first case, it is necessary to supply enough energy to weaken the interactions within the Na^+^ and Cl^−^ lattice, which requires a temperature as high as 801 °C [75]. Beyond this temperature, NaCl transitions into a liquid state. It is only at such a high temperature that the ionic bonds stabilizing the crystal lattice can be overcome. It is worth noting that the melting point of silver is 961.8 °C, while most brass alloys melt in the range of 850–950 °C [76]. This demonstrates the significant amount of energy needed to melt inorganic crystals with ideal ionic bonding, such as ordinary NaCl.

The second way to destroy the crystal lattice is by dissolving the crystal [77]. The solvent must have the ability to minimize the strong ionic bond between the cations and anions. Water serves as an ideal solvent due to its dipolar nature, which allows it to surround ions in a manner that gradually weakens the bonds [78]. In the solvation process (referred to as hydration in the case of water) 4 to 6 water molecules are required to form the primary hydration shell around sodium cations. This shell is crucial for transferring Na^+^ from the lattice into the solution. Similarly, chloride anions must be surrounded by water molecules to shield their negative charge. Water thus acts as a medium that weakens ionic bonds, enabling salts like NaCl to dissolve. As water is gradually removed from the medium, the ions draw closer together, their interactions intensify, and the orderly reconstruction of the crystal lattice with strong ionic bonds occurs.

**Figure 5:**
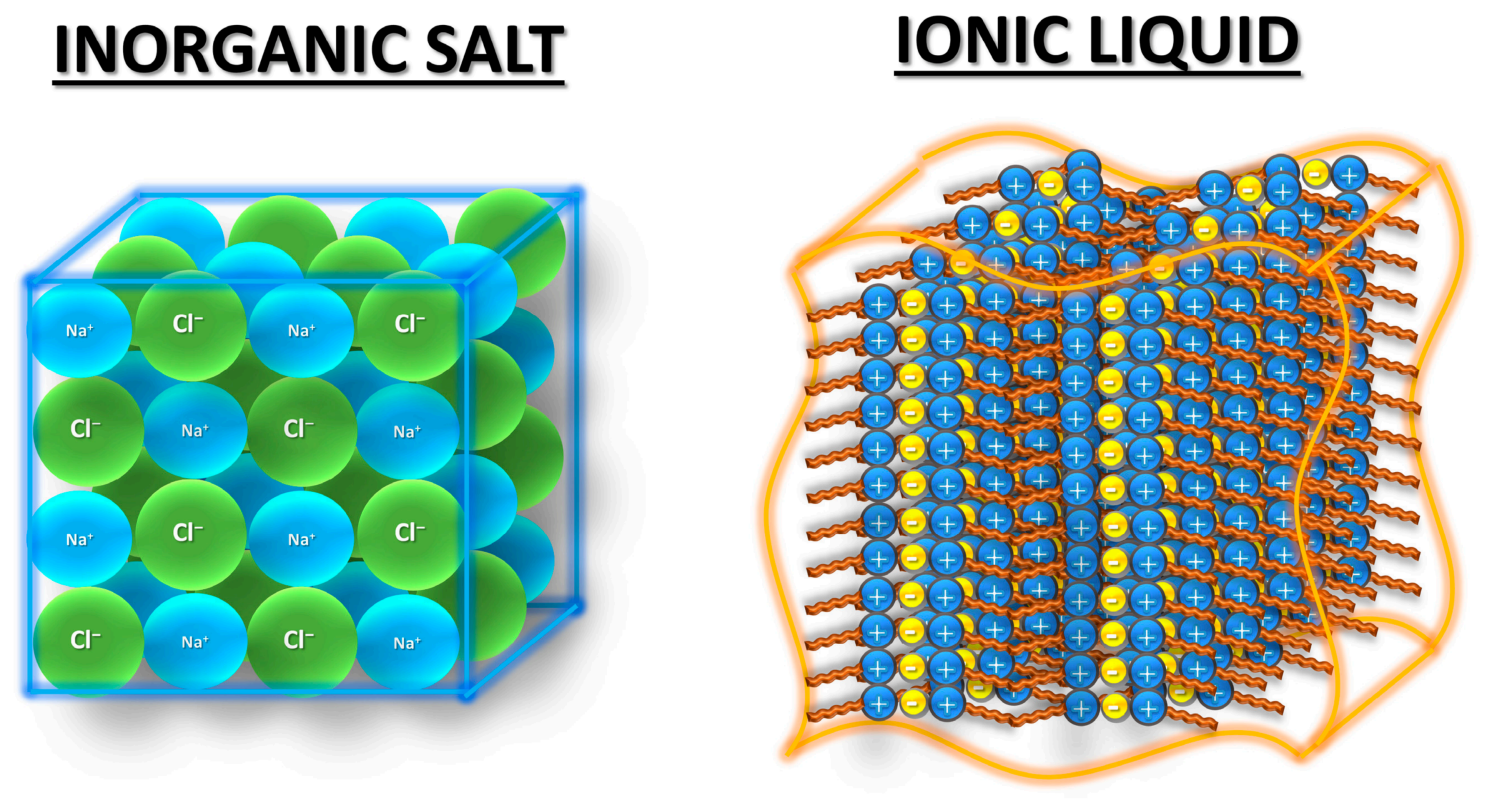
Schematic comparison of the structural organization of a conventional ionic crystal lattice and an ionic liquid.

In fact, the behavior of ILs should be analyzed from a similar perspective while taking into account their unique structural characteristics. Unlike conventional inorganic salts, ionic liquids contain bulky and often asymmetric organic ions with heterogeneous charge distribution and, in many cases, hydrophobic molecular fragments. These structural features distinguish aqueous ionic liquid systems from conventional inorganic salt solutions and influence not only crystal packing but also hydration and ion-pairing behavior. As illustrated in Figure 5, the larger size and structural complexity of organic ions hinder efficient packing compared with the highly ordered crystal lattice of NaCl. Consequently, the interactions responsible for lattice stability are weaker, facilitating the formation of liquid phases at substantially lower temperatures than those observed for conventional inorganic salts [79,80,81]. Furthermore, whereas conventional inorganic salts typically undergo extensive hydration and dissociation in water, the extent of hydration and ion association in ionic liquids depends strongly on the chemical structure of the constituent ions, particularly their size, molecular asymmetry, and hydration ability. These differences should be taken into account when comparing the environmental behavior of aqueous ionic liquid systems with that of conventional inorganic salt solutions.

Larger organic ions and greater lattice distortions further reduce the energy required to disrupt lattice order. This distinction allows ILs to be classified into subcategories, such as room-temperature ILs [11,82] and low-melting ILs [83,84]. More importantly, the relatively weak and highly distributed ionic interactions responsible for their liquid state may also influence their behavior in the presence of water.

### 2.2. What Are Ionic Liquids?

The most important of these is the non-chemical definition of ILs, which is based solely on their physical state at temperatures equal to or below 100 °C [82,85]. Even in geology, a mineral is defined as a substance with a crystal structure, which is why amber is not considered a mineral, whereas graphite and diamond certainly are [86]. In contrast, ILs do not necessarily have to possess a crystal structure. This definition may have several important implications when interpreting the behavior of ILs under environmental conditions.

First, water can be incorporated into the crystal lattice of inorganic salts. For instance, water molecules are coordinated by copper ions in copper sulfate [87]. Can water similarly integrate into the crystal lattice of ILs? This raises an important question because, in the majority of studies on ILs, the existence and possible structure of their crystal lattice have not been analyzed. Discussions of their physicochemical properties often begin with the production of oily residues, with only sporadic determination of water content, for example, using the Karl Fischer method [88]. Moreover, if other solvents are used in the crystallization process, it is possible for solvent molecules to similarly become incorporated into the crystal structure [89]. 

The absence of a requirement to form a well-defined crystal with a specific lattice structure has occasionally resulted in the term “IL” being applied to incompletely purified reaction mixtures or insufficiently characterized products. Such practices may complicate comparisons between studies and contribute to inconsistencies in data interpretation. When looking at the interactions within organic–inorganic or organic–organic ionic networks not only ionic interactions but also interactions associated with hydrophobic and hydrophilic molecular fragments must be taken into account. Any structure rich in electrons, such as double bonds, aromatic rings, or lone electron pairs, contributes its unique influence to the interactions of such particles within potential crystal lattice. These include among others a combination of Coulomb forces, hydrogen bonding, π-π interactions, and dispersion forces [90]. This also applies to hydrophobic fragments, such as alkyl chains.

This raises the question of which interactions dominate in a given IL. At the extremes, one may envision systems in which ionic interactions largely determine physicochemical behavior and water solubility, as well as systems in which hydrophobic molecular fragments exert a much stronger influence on the observed properties. Most ILs likely fall somewhere between these two conceptual limits. Nevertheless, consideration of these two extremes is useful for illustrating how different interactions may influence their behavior. The first of these groups, water-soluble ILs, is relatively predictable in terms of its physicochemical properties [91]. When dissolved in water, the ions are independently solvated by water molecules. Given that other ions are always present in aqueous solutions, the system becomes a mixture of ions.

### 2.3. Do All Ionic Liquids Dissociate to the Same Extent?

One important question that has received surprisingly little attention is whether all ionic liquids dissociate to the same extent after entering aqueous environments. This issue is particularly relevant because, although environmentally oriented studies have primarily focused on ionic liquids containing hydrophilic functional anions, many industrially important ionic liquids incorporate weakly hydrated anions such as PF_6_^−^, BF_4_^−^ and Tf_2_N^−^.

The available evidence suggests that conclusions drawn for ionic liquids containing hydrophilic anions should not be automatically extended to all ionic liquid classes. Computational studies together with physicochemical experiments indicate that ionic liquids containing weakly hydrated anions exhibit a greater tendency toward ion association than systems containing strongly hydrated anions [92,93,94,95]. Rather than electrostatic interactions alone, the hydration ability of the anion appears to play a dominant role in determining the balance between ion–ion and ion–water interactions. Consequently, ionic liquids containing weakly hydrated anions may retain a higher degree of ion pairing in aqueous media than ionic liquids containing hydrophilic functional anions.

However, these conclusions should be interpreted with caution. The currently available evidence originates predominantly from molecular simulations and simplified physicochemical studies, whereas direct experimental investigations performed under environmentally relevant conditions remain scarce. Consequently, although existing studies suggest that ionic liquids containing hydrophobic anions may behave differently from systems containing hydrophilic functional anions, the environmental significance of these observations remains largely unexplored. Additional studies performed under realistic environmental conditions are therefore needed before these findings can be generalized across different classes of ionic liquids.

### 2.4. To What Extent Do Ionic Liquids Behave as Surfactants?

As the structural complexity of organic ions increases, their unique physicochemical characteristics become more pronounced. A representative example is provided by cationic surfactants. These compounds contain a positively charged head group and a hydrophobic alkyl chain that exhibits limited affinity for the aqueous phase. To minimize energetically unfavorable interactions with water, the hydrophobic chains tend to associate with one another, leading to the formation of micelles and other supramolecular structures. Similarly, the hydrophobic tail of a cationic surfactant can interact with biological membranes by inserting into their lipid bilayer. Since the interior of cell membranes is hydrophobic, these interactions are primarily driven by van der Waals and other non-covalent forces. When microorganisms are exposed to solutions containing cationic surfactants, the hydrophobic tail tends to penetrate the membrane interior, while the positively charged head group interacts with the membrane surface and disrupts the organization of membrane phospholipids. This process may ultimately compromise membrane integrity and lead to cell death. The antibacterial activity of cationic surfactants is therefore closely linked to their ability to interact with biological membranes and is illustrated schematically in Figure 4.

**Figure 6:**
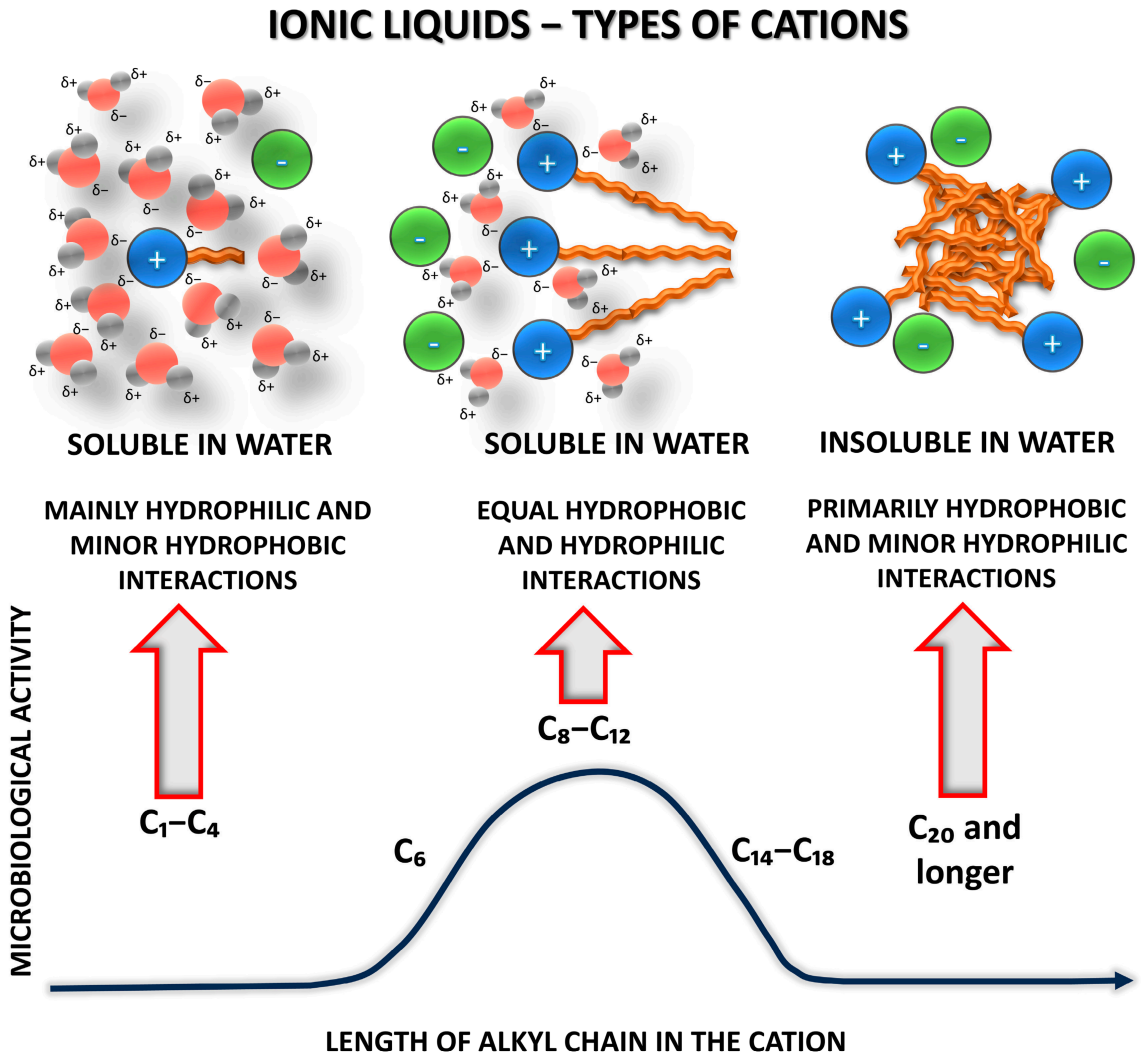
Relationship between alkyl chain length, water solubility, and biological activity of quaternary ammonium compounds. In microbiological literature, this phenomenon is commonly referred to as the cut-off effect. Since quaternary ammonium cations constitute the majority of cations used in ionic liquid synthesis, this relationship is directly relevant to understanding the behavior of many ILs. Notably, the observed trend is largely independent of whether the accompanying anion is organic or inorganic.

It is worth noting that antimicrobial activity is strongly influenced by alkyl chain length and the associated degree of hydrophobicity. Short alkyl chains generally exhibit insufficient hydrophobicity to efficiently interact with biological membranes. Conversely, excessively long alkyl chains may substantially reduce water solubility, limit biological availability and reduce antimicrobial activity. As a result, only cationic surfactants possessing hydrophobic chains of appropriate length exhibit maximum biological activity. This phenomenon is commonly referred to as the cut-off effect and describes the existence of an optimal alkyl chain length that maximizes activity against microorganisms. Importantly, the dependence of biological activity on alkyl chain length suggests that hydrophobic molecular fragments may exert a stronger influence on observed biological effects than the presence of ionic charges alone. This relationship is illustrated in Figure 6.

Besides differences associated with alkyl chain length, experimental studies have shown that ionic liquids representing different cation families, including imidazolium-, quaternary ammonium-, and phosphonium-based ionic liquids, generally exhibit similar membrane interaction mechanisms involving adsorption at the membrane surface followed by insertion of the organic cation into the lipid bilayer [96,97]. Although the cation generally plays the dominant role in membrane perturbation, experimental evidence suggests that the nature of the anion may also influence membrane organization by modifying lipid packing or promoting local bilayer defects [98].

These observations raise the question of whether ILs should be regarded as a distinct class of surfactants or whether many of their surfactant-like properties arise from mechanisms already well established for conventional cationic surfactants. Based on the known mechanisms governing interactions between cationic surfactants and biological membranes, antimicrobial activity requires both water solubility and a hydrophobic molecular fragment capable of interacting with membrane lipids. The hydrophobic fragment cannot be excessively short or excessively long. If it is too short, hydrophobic interactions are insufficient to promote membrane association and the formation of supramolecular structures such as micelles. As alkyl chain length increases, hydrophobic interactions promote the formation of organized structures that are well documented in colloid and surface chemistry [99]. Beyond a certain level of hydrophobicity, however, water solubility decreases substantially, limiting biological availability and reducing activity.

**Figure 7:**
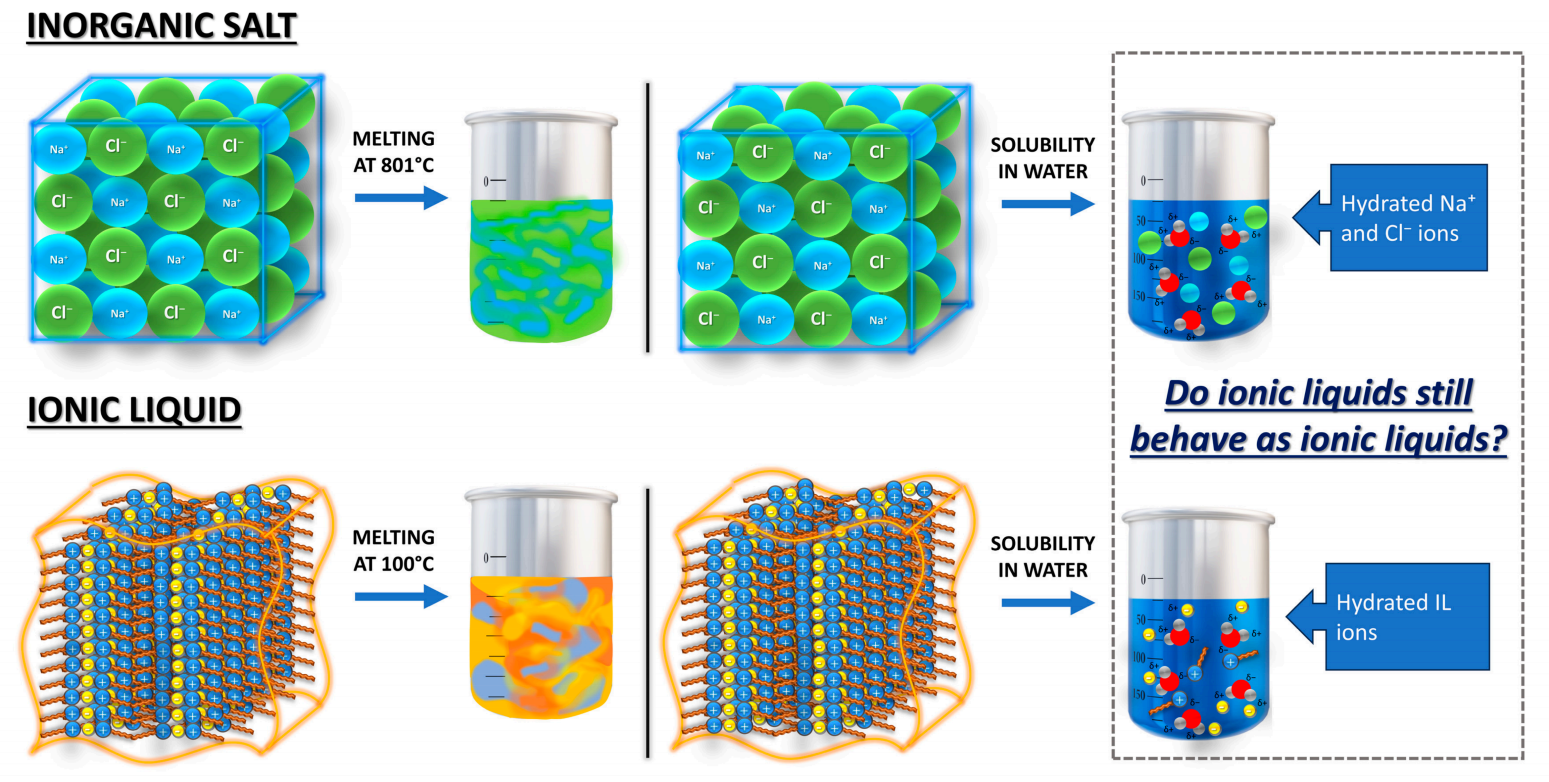
Conceptual comparison of ion hydration in inorganic salts and ionic liquids following dissolution in water.

In all of these scenarios, the behavior of the cationic compound is fundamentally governed by its interactions within an aqueous environment. Water is therefore not merely a medium but a critical factor determining the observed properties. This is particularly relevant because the conventional definition of ILs is based primarily on physical state and does not explicitly consider interactions with water. If aqueous solutions of ILs are still regarded as ILs, it becomes reasonable to ask whether aqueous solutions of conventional inorganic salts should be interpreted in an analogous manner. This conceptual dilemma is illustrated in Figure 7. Although inorganic salts and ionic liquids differ fundamentally in ion size, molecular asymmetry, charge density, amphiphilic character, and solvation behavior, increasing water content promotes ion solvation in both systems and changes the balance between ion–ion and ion–water interactions.

This interpretation may lead to an interesting conceptual consequence. If aqueous solutions of ILs are considered ILs, then similar reasoning could potentially be extended to aqueous solutions of conventional inorganic salts, as all water-soluble salts form liquid solutions within the temperature range commonly used to define ILs. While such a classification is clearly not intended, it illustrates the ambiguity that may arise when physical state alone is used as the primary defining criterion. Furthermore, dissolved inorganic salts are also capable of modifying the physicochemical properties of water. One well-known example is boiling point elevation, whereby dissolved ions increase the boiling point of the solution.

### 2.5. Influence of Cations on Anion Properties

Building on these fundamental principles, some researchers have proposed that the appropriate selection of cations may influence the properties of anions, likely due to ion pair formation. However, the extent to which such interactions persist under different environmental conditions is not always explicitly considered. For instance, research by [100] investigated the influence of cations on the crystalline structure and behavior of cation–anion pairs, focusing on imidazolium-based surface-active ILs with varying alkyl chain lengths. Their findings indicated that increasing alkyl chain length enhanced surface activity and hydrophobicity [101,102]. These observations may also be interpreted in the context of well-established relationships between molecular structure and physicochemical properties, where increasing alkyl chain length is known to increase hydrophobicity and surface activity [103,104]. Consequently, it remains important to distinguish between effects arising from ion pair interactions and those associated with the intrinsic properties of the constituent ions.

Given the emphasis placed on the unique properties of ILs in the literature, it is important to move beyond indicative observations and pursue experimental validation under environmentally relevant conditions [105]. One key factor influencing IL behavior is the presence of water, as anhydrous systems may exhibit substantially different characteristics from ILs introduced into aqueous environments [103,106]. This distinction is particularly relevant when considering the stability, persistence, and mobility of ion pairs in complex matrices such as soils, sediments, and biological systems [105,107].

### 2.6. Independence of Cation and Anion Behavior

A particularly relevant example of potential ionic interactions is the attempt to control the mobility of herbicidal anions, such as phenoxyacetic and benzoic acid derivatives (e.g., 2,4-D and dicamba), by pairing them with cations of increasing hydrophobicity [108]. Theoretically, such herbicidal ILs were expected to reduce herbicide mobility in soil, limiting leaching and enhancing efficacy while simultaneously reducing the required application rates.

Several experimental studies have investigated whether herbicidal ionic liquids remain associated as stable ion pairs under environmentally relevant conditions. This question is particularly important because environmental studies on conventional ionic liquids have repeatedly demonstrated that sorption and mobility are strongly influenced by the surrounding matrix and the physicochemical properties of the individual ions rather than solely by the identity of the ionic liquid itself [103,105,107]. These studies examined how these cations properties influence the sorption and mobility of ILs containing dicamba [108,109,110] and 2,4-D anions [108,111,112] in aquatic and soil environments. Phenoxy acids such as 2,4-D and MCPA, as well as benzoic acid derivatives such as dicamba, are known for their high mobility and short half-life in the soil. As a result, they are quickly leached, which necessitates the use of higher doses to ensure the effectiveness of the treatment. Therefore, modifications that enhance their retention would be highly desirable from both a user perspective and an environmental standpoint.

However, 2-D NMR analyses and molecular modeling provided no evidence for persistent cation–anion interactions under the investigated conditions. The results indicated that cation sorption occurred independently of the anion and was primarily influenced by cation hydrophobicity. The dominant role of cation hydrophobicity in controlling sorption is consistent with previous studies on conventional ionic liquids, where sorption was shown to depend primarily on cation structure and soil properties [107,113]. Meanwhile, the 2,4-D anion showed no sorption in any of the analyzed systems, regardless of the cation used. Similarly, dicamba anions were not retained in any tested IL systems, whether in OECD-standardized or real agricultural soils.

**Figure 8:**
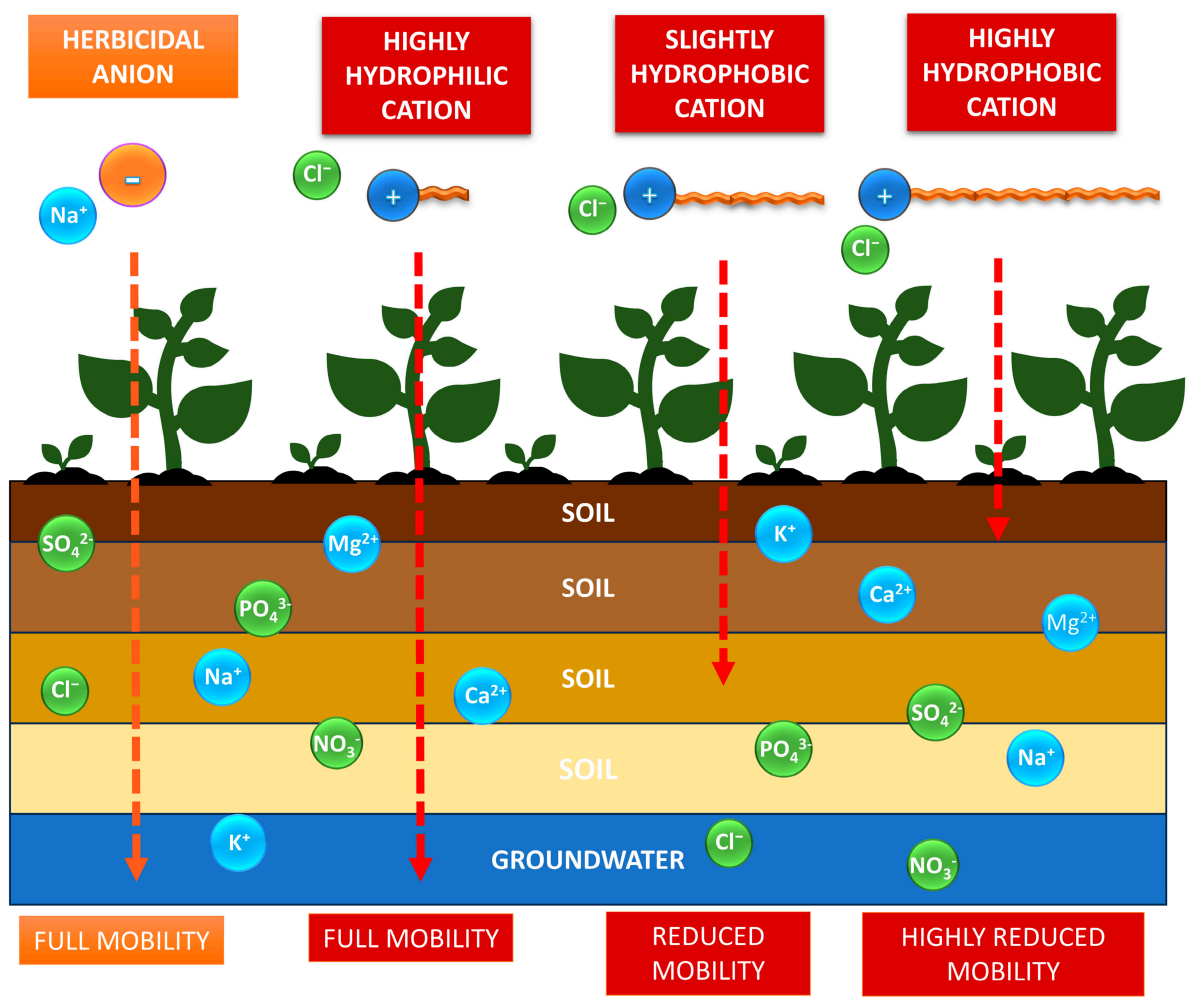
Conceptual illustration of the independent environmental behavior of herbicidal anions and hydrophobic cations in soil environment, based on experimental evidence.

Overall, the available experimental evidence indicates that none of the examined cations restricted the mobility of dicamba or 2,4-D anions in soil. These observations suggest that, under the investigated environmental conditions, cations and anions largely behaved independently rather than as stable ion pairs (Figure 8). This is expected, given that soil contains various competing ions are readily solvated by water and contribute to a complex ionic environment [105,106,107].

Consequently, the absence of observable interactions in NMR spectra for pure ILs cannot be considered evidence of ion pairing under environmental conditions, where solvation and interactions with surrounding ions dominate [103,105]. These findings support the view that ILs introduced into environments may often behave as dissociated ions rather than as persistent cation–anion assemblies.

An alternative and well-established approach to influencing mobility and enhancing herbicide penetration into plant tissues involves the incorporation of surfactants. This strategy has been extensively documented and is widely applied in commercial herbicide formulations, particularly through the addition of nonionic surfactants [114]. The effectiveness of this method is further supported by the fact that nonionic surfactants exhibit significantly lower soil sorption and, crucially, pose substantially less environmental risk compared to cationic surfactants. However, this strategy fundamentally differs from the concept of designing ILs for controlled herbicide behavior. 

Despite these well-documented alternatives, numerous studies have sought to explore the potential of hydrophobic cations to modulate the mobility of herbicidal anions, such as bentazone [115], clopyralid [116], dicamba [117], mesotrione [118] or picloram [119]. However, direct evidence supporting the proposed cation–anion interactions was frequently limited. In many cases, studies reported reduced mobility of ILs containing long-chain hydrophobic cations yet provided limited direct evidence of interactions between cations and anions that would justify this effect [107,113]. The absence of comparative assessments with precursor salts further complicates interpretation, as the observed changes in mobility may stem solely from the physicochemical properties of individual cations rather than from intrinsic modifications of the IL system itself. Therefore, attributing shifts in mobility exclusively to ion pairing without appropriate controls and systematic analysis remains difficult to justify. 

Since mobility is inherently linked to sorption dynamics within the soil matrix, a comprehensive understanding of cation and anion interactions in this context is essential. Therefore, additional studies have been conducted on ILs containing glyphosate anions [119]. Glyphosate is recognized for its relatively long half-life in the soil, primarily due to its moderate sorption to soil particles. In these experiments, glyphosate sorption was relatively high in both model and agricultural soils, yet it remained unaffected by the type of hydrophobic cation used. Hydrophobic cations were almost entirely adsorbed by the soil with minimal leaching, while hydrophilic cations showed only partial adsorption. These results further support the observation that glyphosate sorption was largely independent of cation selection under the investigated conditions and provide limited support for assumptions regarding synergistic effects arising from persistent ion pairs in ILs. Collectively, these findings suggest that the hydrophobic nature of the cation had little influence on the sorption behavior and mobility of the investigated herbicidal anions. 

Moreover, other comprehensive sorption studies on various ILs, where the adsorption of organic cations was the primary focus, have consistently reported that the efficiency of this process is directly correlated with alkyl chain elongation (reflecting increased hydrophobicity) as well as with specific soil properties [107,113]. In these studies, no clear influence of the anion on this interaction was observed, supporting the view that cations and anions may behave largely independently under environmentally relevant conditions [62,120,121]. Independent studies employing chloride salts further supported the observation that cations undergo sorption in soil, with the magnitude of this process dictated predominantly by alkyl chain length [122,123]. The fundamental nature of cationic interactions appeared unaffected by their pairing with herbicidal anions, further supporting the interpretation that sorption behavior was primarily governed by cation properties.

These observations have also been reported for imidazolium-based ionic liquids [124], which examined imidazolium-based ILs and suggested that the contribution of the anion to sorption dynamics was limited. Instead, the governing factors were the physicochemical properties of the cation, particularly its hydrophobicity, which modulates intermolecular interactions such as van der Waals forces and hydrogen bonding, ultimately shaping the sorption behavior in complex environmental matrices.

As mentioned earlier, in aqueous solutions, the cations and anions of ILs are solvated and behave independently of each other, while their environmental behavior is further modified by interactions with surrounding ions and the physicochemical properties of the matrix [97,98,99]. Additionally, other ions present in the soil can stabilize the separate migration of ions. The expected behavior may therefore resemble that observed for mixtures of conventional inorganic salts. For example, the nitrates of nickel, copper, and lead can form solutions, whereas the addition of NaCl may reduce the precipitation of lead chloride due to its lower solubility under these conditions. Similarly, precipitation phenomena involving IL constituents may occur, although this aspect requires further investigation. Consequently, the hypothesis that herbicide mobility can be controlled through IL design warrants further investigation and critical reassessment, particularly under environmentally relevant conditions that account for the complexity of soil and aquatic matrices [104,105,125].

### 2.7. Biological Activity as an Indicator of Ion Pair Stability

The integrity of ILs can also be evaluated through toxicity studies that assess their effects on microorganisms. This approach is relevant because previous studies on conventional ILs have shown that biological activity is strongly related to molecular structure, particularly the type of cation and the length of the alkyl substituent. In general, increasing alkyl chain length increases hydrophobicity and is frequently associated with higher toxicity toward microorganisms and other test organisms, although the influence of the anion may vary depending on the tested system [96,101,126,127].

Antimicrobial activity tests were conducted using ILs containing glyphosate anions against enrichment cultures [128] and standard microorganisms, including *Escherichia coli*, *Staphylococcus aureus*, and *Candida albicans* [120]. These studies also included chloride and bromide salts of hydrophilic and hydrophobic cations, identical to those in the tested ILs. The results indicated that the glyphosate anion exhibited little or no toxicity toward the tested microorganisms. In contrast, toxicity was primarily observed in experiments where microorganisms were exposed to hydrophobic compounds, which behaved similarly to typical cationic surfactants. These findings suggest that the observed toxicity was largely associated with the properties of the cations present in the investigated ILs.

Similar conclusions have been reported across studies investigating ILs containing other herbicidal anions, such as 2,4-D and MCPA [129], MCPP [29], and dicamba [29,104], tested against *Pseudomonas putida*, *Pseudomonas aeruginosa*, *Staphylococcus aureus*, and *Candida albicans*. Collectively, these studies indicate that increasing alkyl chain length was directly correlated with increased toxicity and appeared to be the dominant factor influencing antimicrobial activity. This interpretation is consistent with independent studies showing that longer alkyl chains in IL cations are generally associated with stronger toxic effects, including effects on bacteria, aquatic organisms, and soil microorganisms [101,115,116,117]. The low toxicity of herbicidal anions further supports the interpretation that the hydrophobic cation was primarily responsible for the toxicity observed in both ILs and their chloride counterparts. 

In certain microbial toxicity studies, such as those by [130,131,132,133,134,135,136,137,138], only the ILs with herbicidal activity were evaluated, without considering the impact of the precursor salts used in their synthesis. In other cases, various ILs were investigated in which both the cation and anion exhibited toxicity. However, an important limitation of these studies was the lack of separate toxicity assessments for individual precursors. Consequently, it remained unclear whether the increased toxicity observed against microorganisms was a cumulative effect, representing a simple summation of individual precursor toxicities, or whether it resulted from newly acquired, unique properties of the synthesized IL [139,140,141]. As a result, toxicity was attributed to the entire compound without a rigorous assessment of whether the primary determinant was the hydrophobic cation, the anion, or their potential synergistic interaction. This limitation is important because broader toxicity studies have repeatedly shown that both cation and anion identity can influence biological effects, while cation structure and hydrophobicity often remain dominant factors [101,106,115].

Results indicating the absence of significant interactions between cations and anions have also been reported for other classes of ILs. Studies on imidazolium- and pyridinium-based ILs against microorganisms such as *Escherichia coli*, *Staphylococcus aureus*, *Bacillus subtilis*, *Pseudomonas fluorescens*, and *Saccharomyces cerevisiae* [142] consistently indicated that the anion within the IL structure exerted little observable influence on toxicity. Instead, the principal determinant of toxicity was the progressive elongation of the alkyl chain in the cation, which enhanced hydrophobicity and, consequently, cytotoxic effects. Similar relationships between chemical structure and toxicity have also been described in broader ecotoxicological studies, including test batteries covering aquatic and terrestrial organisms [104,106,143]. 

Particularly interesting findings were presented in [144], which assessed the toxicity of imidazolium-based ILs toward *Daphnia magna* and *Photobacterium phosphoreum*. This study directly compared the toxic effects of chloride salts and their corresponding ILs. The results indicated that, regardless of whether a precursor salt or an IL containing the same cation was used, the toxicity outcomes remained comparable. Similar trends were observed in other studies assessing the toxicity of ILs and chlorides against *Photobacterium phosphoreum* [145]. The increased toxicity of the tested compounds was found to be closely linked to structural parameters governing hydrophobicity, rather than to the combination of a given cation with a different anion. This is consistent with aquatic toxicity studies showing that IL toxicity toward organisms such as *Daphnia magna*, algae, and bioluminescent bacteria is strongly affected by cation structure and alkyl chain length [103,143,146].

From a design perspective, if ILs were to acquire substantially different biological properties through cation and anion interactions, the combination of a non-toxic anion with a toxic cation might theoretically reduce the toxicity associated with the cation. However, the reported results did not indicate lower toxicity compared with the corresponding chloride salts. These findings support interpretation that toxicity was primarily associated with the cation and that no evidence of stable interactions between cations and anions affecting toxicity was observed under the investigated conditions. Consequently, toxic cations appear to retain their biological activity independently of the accompanying anion, whether chloride or herbicidal, when dissolved in aqueous environments.

This provides additional evidence that the observed toxic effects are closely linked to the behavior of cations acting as surfactant-like compounds. Since the activity of surfactants is inherently associated with aqueous environments, the role of water becomes central when interpreting the behavior of ILs. Therefore, the classification of highly diluted aqueous IL solutions as ILs warrants further discussion.

Based on the previously discussed reports on individual cations and anions, further investigations were carried out to examine three distinct systems: (1) ILs containing glyphosate and 2,4-D anions paired with hydrophobic cations, (2) precursor salts used in the synthesis of these ILs, and (3) mixtures of these salts without undergoing synthesis [147,148]. These systems were analyzed for their sorption behavior including interactions with microplastics, toxicity against *Pseudomonas putida* using the *trans*/*cis* ratio of unsaturated fatty acids as a biomarker, and biodegradability.

**Figure 9:**
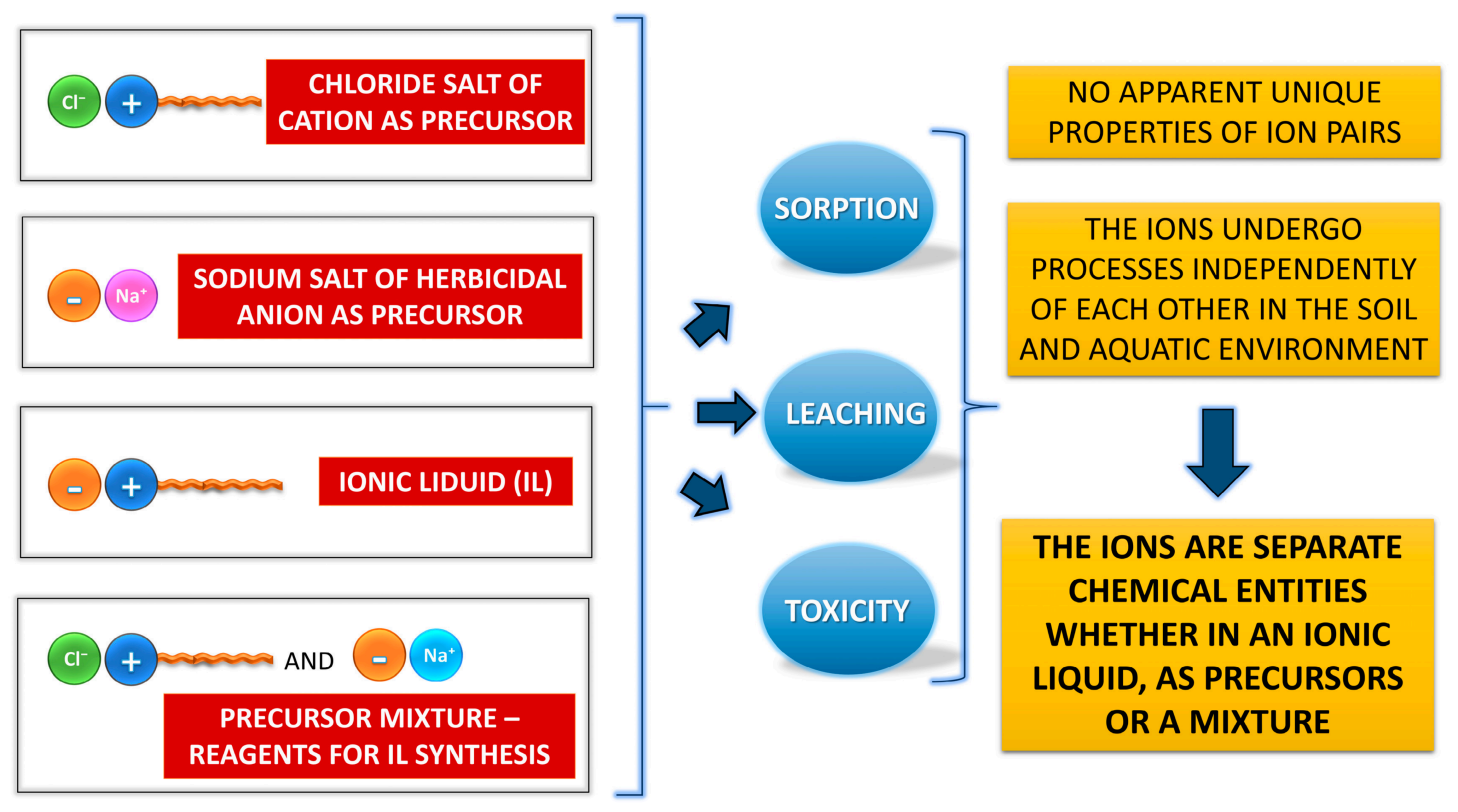
Conceptual comparison of the environmental behavior of herbicidal ionic liquids and their corresponding precursor compounds.

The results showed that, regardless of the system tested, sorption, toxicity, and biodegradability were primarily governed by the individual properties of the constituent ions (Figure 9). Notably, while hydrophobic quaternary ammonium cations exhibited strong sorption onto microplastic particles, herbicidal anions showed little or no retention on these surfaces, consistent with their high mobility in aqueous systems. This behavior aligns with previous observations in soil environments, where the retention and toxicity of IL cations are strongly affected by hydrophobicity, soil organic matter, clay minerals, and microbial community responses [101,107,113,114]. Collectively, these findings are consistent with the interpretation that cations and anions may behave largely independently in environmental matrices, with no evidence of persistent ion pair formation under the investigated conditions.

### 2.8. Environmental Implications for the Design of Ionic Liquids

The findings of these multifaceted studies are best summarized by the results of experiments comparing the mineralization of ILs derived from ^13^C-labeled surface-active cations and ^13^C-labeled MCPA anion in aqueous environments and agricultural soil from a typical farmland [149]. These experiments complement previous studies demonstrating that the environmental fate of ionic liquids is determined by a combination of molecular structure, sorption processes, microbial accessibility, and environmental conditions rather than by a single physicochemical property [103,105,107]. The use of isotope labeling allowed the demonstration of preferential cation degradation by activated sludge, while herbicide mineralization remained negligible. Regardless of whether the cation was introduced as a chloride salt or as an IL with the herbicide, microorganisms utilized the cation as a carbon source. This was confirmed by the ^13^C enrichment of fatty acids extracted from cell membranes. Such observations are consistent with previous biodegradation studies showing that cation structure largely determines microbial utilization of ionic liquids, whereas the accompanying anion generally plays a less pronounced [150,151].

In the soil environment, no cation mineralization was observed, irrespective of whether it was introduced as a chloride or an IL. This was attributed to strong adsorption in the soil, which rendered the cation unavailable to microorganisms, preventing its utilization as a carbon source. A similar relationship between sorption, reduced bioavailability and biodegradation has previously been described for conventional imidazolium ionic liquids in soils [107,113]. In contrast, the MCPA anion underwent mineralization, as evidenced by ^13^C enrichment in fatty acids extracted from cell membranes. These results suggest that the degradation of IL cations and anions proceed independently under environmental conditions. The observed mineralization patterns provide limited evidence for the persistence of stable ion pairs during biodegradation processes.

**Figure 10:**
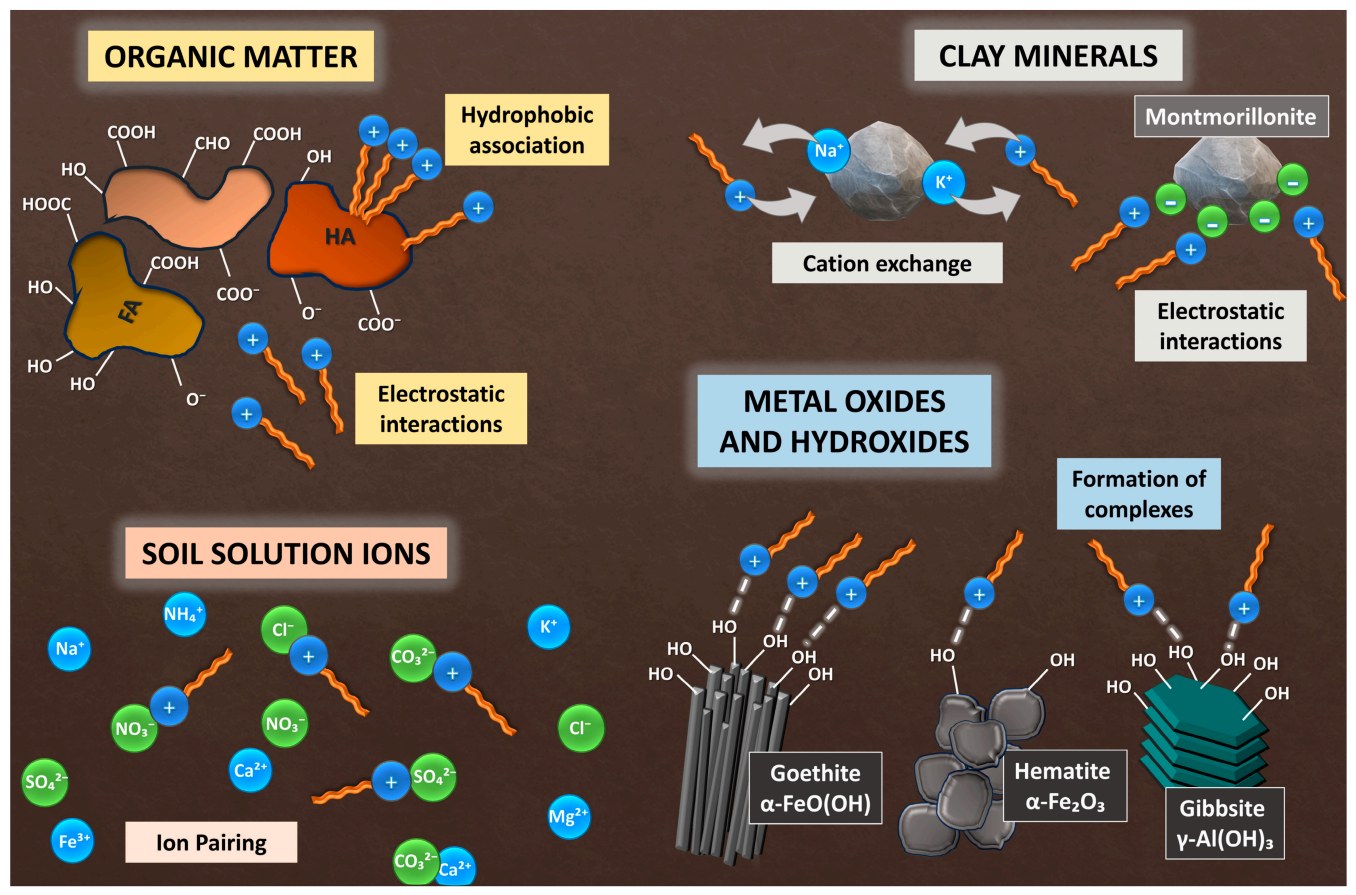
Conceptual illustration of the major interactions governing the behavior of ionic liquid cations in soil environments.

**Figure 11:**
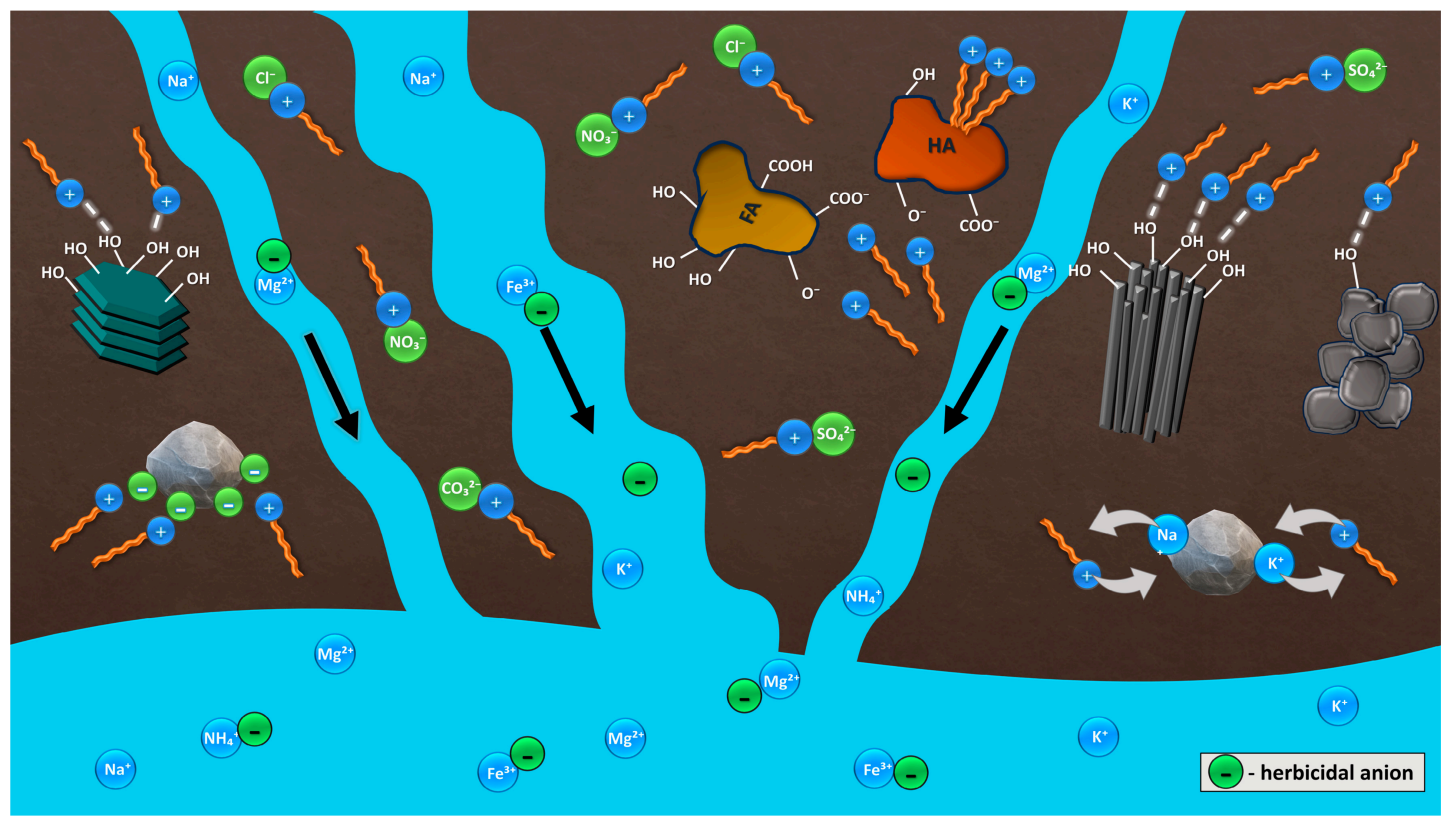
Conceptual model summarizing the environmental behavior of ionic liquids containing a hydrophobic cation and a hydrophilic anion under environmentally relevant conditions.

A crucial aspect often overlooked in studies on ILs is the omnipresence of inorganic salts (cations and anions) in both aquatic and soil environments (Figure 10 and Figure 11). Ionic liquids are introduced into complex environmental matrices rather than pure aqueous systems, and therefore their environmental behaviour should always be interpreted in the context of naturally occurring ions, dissolved organic matter and mineral surfaces [105,107,152]. Seawater typically contains between 35 and 40 kg of dissolved salts per cubic meter [153], while high-quality drinking water holds about 0.5 kg [154], and agricultural irrigation water should not exceed 2 kg [155]. Similarly, soils naturally contain salts, with their concentration in soil leachates ranging from 1 to 12 g/L in slightly to moderately saline soils.

Consequently, ionic liquids introduced into environmental systems are surrounded by a complex ionic matrix rather than pure water. Natural waters and soil pore waters contain dissolved ions, including e.g., Na^+^, Ca^2+^, Mg^2+^, Cl^−^ and SO_4_^2−^. They participate in solvation and electrostatic interactions, thereby influencing the chemical environment in which ionic liquid ions are present. In addition to inorganic ions, dissolved organic matter and mineral surfaces continuously compete for electrostatic interactions and sorption sites, further increasing the complexity of environmental systems [106,107]. Although increasing ionic strength may affect the extent of ion association, direct experimental evidence regarding its influence on ion pairing of ionic liquids under environmentally relevant conditions remains limited, particularly in complex systems such as seawater and saline soils. This represents one of the remaining knowledge gaps in understanding the environmental chemistry of ionic liquids [125].

Beyond simple salt content, a more relevant parameter in soil chemistry is cation exchange capacity (CEC), which reflects the ability of soil particles to retain cations [156]. Previous studies on conventional ionic liquids have similarly demonstrated that cation exchange capacity, together with soil organic matter and mineral composition, plays a key role in governing the sorption and environmental behavior of ionic liquids in soils [101,107,113]. This means that once introduced into soil, ILs, particularly their cations, will undergo exchange with pre-existing cations or other positively charged species present in soil water. Unlike in aquatic environments, where ILs readily dissociate and mix with other ions, their mobility and retention in soil depend on additional factors, including organic matter content, clay mineral composition and soil pH. These environmental factors have also been recognized as important determinants of ionic liquid mobility and bioavailability in terrestrial environments [103,105]. Hydrophobic cations, in particular, tend to be retained more strongly because of their affinity for organic matter and hydrophobic sorption sites (Figure 10).

Consequently, it may be difficult to expect ILs to remain as stable ion pairs under environmentally relevant conditions. Instead, they are likely to interact dynamically with the ionic matrix of both water and soil. The environmental fate of ionic liquids is governed by interactions with complex environmental matrices rather than by a single physicochemical process [152]. Nevertheless, their surface activity, adsorption tendencies, and potential interactions with soil components will continue to influence their environmental behavior. This may help explain why few significant differences have been observed when introducing cations as chlorides, as ILs, or even as mixtures of their precursor salts. Under such conditions, environmental interactions appear to depend primarily on the individual properties of the cation rather than on its initial pairing with a particular anion.

## 3. Conclusions

The evidence discussed throughout this article raises an important question regarding how ILs should be interpreted once they are introduced into aqueous and environmental systems. While ILs are commonly described as compounds possessing tunable properties arising from specific cation–anion combinations, the available experimental observations frequently indicate that, under environmentally relevant conditions, cations and anions behave largely as independent species. Sorption, toxicity, biodegradation, and mobility appear to be governed primarily by the physicochemical properties of the individual ions rather than by persistent interactions within the ion pair.

This observation does not diminish the scientific value of IL research. Instead, it highlights the importance of distinguishing between the behavior of pure ILs and the behavior of their aqueous solutions, where water, dissolved salts, and competing environmental interactions become dominant. The transfer of concepts developed for anhydrous systems directly to environmental conditions may therefore require greater caution and experimental verification.

The studies discussed in this article consistently demonstrate that hydrophobic cations largely retain their characteristic surfactant-like behavior, including sorption to environmental matrices and interactions with biological membranes, whereas the fate of anions is primarily determined by their own physicochemical properties. Similar conclusions emerge from studies investigating toxicity, biodegradation, mineralization, and environmental mobility, where evidence for persistent ion-pair-specific effects remains limited.

These observations invite a broader discussion concerning the widely accepted concept of IL designability. While modifying cation and anion structures undoubtedly alters the properties of the resulting compounds, the extent to which these modifications remain relevant under environmental conditions remains an open question. Future studies should therefore place greater emphasis on experimentally verifying the persistence and environmental relevance of cation–anion interactions rather than assuming their stability based on observations obtained in pure ILs or simplified laboratory systems.

Finally, the rapid expansion of IL research has generated a substantial body of literature describing unique and tunable properties of these compounds. While this progress has significantly advanced the field, the continued repetition of concepts that have not been directly verified under environmentally relevant conditions may complicate data interpretation and hinder the development of a consistent understanding of IL behavior. A more critical evaluation of these assumptions may not only improve experimental design and interpretation but also help define more clearly when ILs truly behave as ILs, and when they are more appropriately considered as mixtures of independently acting ions.

## 4. Review Methodology

This narrative review focuses on the environmental behavior of ionic liquids, with particular emphasis on the environmental relevance of ion pairing under aqueous and soil conditions. The literature was identified primarily through searches of the Web of Science and Scopus databases, supplemented by Google Scholar for citation tracking and identification of additional relevant publications. Representative search terms included combinations of ionic liquids, ion pairing, ion dissociation, hydration, aqueous systems, soil, sorption, toxicity, biodegradation, environmental fate, cation, anion, herbicidal ionic liquids, and surfactants.

Original research articles and review papers were considered, with particular emphasis on studies investigating environmentally relevant aqueous and soil systems. Representative studies covering different classes of ionic liquids, environmental matrices, and experimental approaches were selected to provide a balanced overview of the available evidence. Priority was given to studies addressing sorption, mobility, toxicity, biodegradation, surfactant-like behavior, and ion pairing under environmentally relevant conditions. Studies focusing exclusively on the physicochemical properties of neat ionic liquids or on technological applications, such as catalysis or extraction processes, were included only where they provided background relevant to the environmental interpretation of ionic liquid behavior. No formal publication date restrictions were applied. The final selection of references was guided by their relevance to the central question addressed in this review, namely whether persistent cation–anion interactions remain environmentally significant after ionic liquids are introduced into environmentally relevant aqueous and soil systems.

## Data Availability

The metadata have been deposited in the RepOD repository and can be accessed via the link: https://doi.org/10.18150/1VXZJG.

## Abbreviations

The following abbreviations are used in this manuscript:

2-D NMR	Two-Dimensional Nuclear Magnetic Resonance
2,4-D	2,4-Dichlorophenoxyacetic
CEC	Cation exchange capacity
ILs	Ionic liquids
MCPA	2-methyl-4-chlorophenoxyacetic
MCPP	MCPP-2-(2-Methyl-4-chlorophenoxy)propionic
NaCl	Sodium chloride
OECD	Organization for Economic Co-operation and Development
